# Jolted, Stewed, and Jumbled: Motion, Discomfort, and the Body in English Coach Travel, c. 1660–1820

**DOI:** 10.1093/shm/hkag034

**Published:** 2026-06-08

**Authors:** Alun Withey

**Keywords:** travel, embodiment, motion sickness, discomfort, eighteenth century, coaches

## Abstract

This article explores the embodied experience of coach travel in England, c. 1660–1820, focusing on motion, discomfort, and sensory engagement. Using letters, diaries, and medical texts, it shows how factors including cramped interiors, violent movement, and oppressive atmospheres produced both physical and psychological unease. Motion sickness was widely discussed but poorly understood, reflecting broader uncertainty about the body in motion. By tracing the material and kinaesthetic entanglements of passengers and vehicles, the article extends work on embodiment and mobility, arguing that coach travel was not mere conveyance but a profoundly embodied encounter that unsettled and reshaped the travelling body.

*A country friend to visit, I, a cit*

*Bespeake my passage in a God-permit.*
^1^

*Th’ impatient coachman warns us to prepare,*

*And, long ere sun-rise, mounts his daily care.*

*Scarcely awake, in dreaming mood I rise*

*Enter the coach, and ope[n] my wond’ring eyes*

*On two old females of the Falstaff size:*

*No choice is left me, so, between the two*

*On each side elbow’d, I am doomed to stew.*

*A nurse, a child, a soldier swell’d with pride,*

*And a fat landlord, fill the other side.*

*Day scarcely dawns, before the rugged road*

*From this to that side jolts the motley load*

*One bedlam coughs, the other scolds and stares*

*The landlord snores, child pukes and soldier swears.*

*Of God-permits, if these the comforts be,*

*My feet, thank heav’n, still can carry me.*
^2^

In the 1790s this poem, titled, *Comforts of a Coach,* was published widely. It reads as a litany of all the potential pitfalls, risks, and challenges of eighteenth-century coach travel, from predawn starts to the atmosphere and smell of the coach interior, violent motion, overcrowding, poor or foolish companions, and sickness. If this is what coach travel is like, concludes the author, I would rather walk. Both the ironic title and humorous nature of the verse speak to an important truth. For many, it was *dis*comfort that marked the experience.

Coach travel expanded through the eighteenth century leading more people to travel more frequently and over longer distances. For many this was no gentle waft through the Georgian countryside: it was a rattling, lurching plunge down tracks and roads that were rough and knotty in good weather, and perilous in bad. Neither was the environment inside the coach conducive to comfort. Passengers spent long hours crammed together in its mephitic atmosphere, enduring the sweating, coughing and farting bodies and twattling chatter of their fellows. At its worst, coach travel threatened a full-scale assault on body and senses. It could be an intensive, disruptive somatic experience, and a deeply embodied form of mobility.

A focus on embodiment has been one of the most recent developments in the history of the body. In the wake of the ‘Material Turn’ (and later the ‘new materiality’) in historical writing since the early 2000s, focus turned to the complex interplay between historical actors and the material worlds they inhabited.^3^ The relationship between the physical body and material objects is now a key framework for understanding the body and identity, and particularly the ways in which bodies and objects co-created identity and experience.^4^ Critics of the ‘new materialism’ however, objected to the subordination of the ‘experiencing body’ and how the praxis of daily life ‘felt’ in the past. In response, historians including Karen Harvey, Ben Jackson, and Matthew McCormack have engaged with embodiment in a variety of ways, with letters and ego documents proving particularly fruitful for exploring the complex dialogue between the material body and embodied experience, particularly in relation to material culture.^5^

The embodied experience of travel has attracted recent attention, with calls for greater engagement with embodied motion and mobility, as well as the lived experience of travel more broadly. Travel is, as Charles Forsdick notes, ‘a profoundly corporeal experience’.^6^ The body is the beginning and end of travel. It is, by turns, an object in motion, a vehicle that moves through time and space, an ‘interface’ between the inner and outer world; it can facilitate or, by its own fallibility, impede travel.^7^ How it actually ‘felt’ to travel, and to be in motion, however, has received less attention. What, for example, were the perceived effects, real or imagined, of movement and different modes of transport, upon past travellers? On ships, on horseback, or in coaches, for example, uniform motion could not be guaranteed and instead was subject to the quality of the sea or road and the caprices of the animal. Less attention has been paid towards involuntary motion, the movement imposed upon the body, rather than *of* or *by* it. Drawing on methodologies from the emerging field of mobility studies, Gerrit Verhoeven’s recent study of embodied mobility uses early modern Netherlandish travel journals to recover the sensory experience of motion on various forms of transport including horseback, carriage and ship.^8^ Since, as Verhoeven notes, the embodied experience of mobility has barely yielded a few pages in Grand Tour historiography there is still much to be said. Indeed, there are broader gaps. As Eva Johanna Holmberg has argued, historians have yet to fully engage with the frailties, both physical and psychological, of early travellers, calling for more work on the minutiae, as well as the meta.^9^ Mark Williams also suggests that the emphasis on ‘success stories’ in travel—the satisfactory journey; the fruitful experience of the journey and destination; the safe return—risks unrealistic representations of the experience of premodern travel.^10^

Seeking to address such gaps this article explores the sensory experience of motion through a case study of coach travel in Britain from the later seventeenth through to the early nineteenth-century. This choice of long periodisation enables consideration of change over time in travelling practices and experiences but also reflects continuities in terms of the persistence in humoral beliefs about the effects of travel upon the body. It is also partly governed by the availability of source materials: the episodic and inherently subjective nature of individual accounts of coach travel defies neat chronology or straightforward narratives of change over time.

This study offers contributions to various fields including the histories of travel, medicine, and the body. First, while the dangers of coach travel, such as accidents and misfortunes suffered by travellers, have received some attention, how coach travel actually *felt* and was experienced by individuals has generally not. A notable exception is that of Yoojung Choi’s study of women’s stagecoach travel through Delarivier Manley’s seventeenth-century account of a journey to Exeter. As Choi notes, women faced both moral and physical challenges in travelling by coach, ranging from the physical discomforts of the journey to the gaze and censure of men.^11^ In particular, the embodiment of *dis*comfort in travel remains under-researched. It might be argued that sea travel offers a more ready and recognisable subject for assessing the effects of motion. Considering domestic as well as foreign travel, however, far more people travelled by coach in the eighteenth century than ever boarded a ship. The coach, therefore, offers a wholly new dimension to the somatic experience of travel and discomfort.

Second, a focus on motion raises questions about how the body was believed to react to, or be affected by, different forms of movement. Coach travel arguably popularised a new form of motion. While people had obviously travelled on horseback and in wheeled carts for millennia, coach travel in its various forms added new dimensions, including technological changes, speed, materiality, and the sensory environment of the vehicle. Medical authors debated the healthiness, or otherwise, of the motion of the coach. Some viewed it as a form of exercise, or noted its relaxing, even soporific, effects. Others, however, viewed it as potentially injurious to health.

Third, it offers a new study of both the supposed causes, and experiences of, motion-induced sickness. Whilst, again, seasickness might seem the more obvious choice, the term ‘coach sickness’ emerged in the eighteenth century both as a term and a specific condition, with its own causal framework. Motion sickness has again received little attention either in travel or medical histories but has much to offer as a key discomfort of travel. Drawing on ego-documents and medical, philosophical, and popular publications, this article charts both ideas about causation and experiences of motion sickness. Amidst the general expansion of leisure travel, the eighteenth century saw much discussion about the precise nature and causes of motion-induced sickness. Whilst uncomfortable, it was a situational reaction to motion, rather than symptomatic of a disease and, as such, was not necessarily medicalised. Instead, it was subsumed within broader, and older, ideas about vomiting and perhaps simply accepted as one of the many inevitable travails that accompanied travel.

The main sources for this study are English travellers’ letters drawn from online databases including ‘Enlightenment Letters’, ‘Social Bodies’, and ‘Curious Travellers’, alongside travel narratives and accounts, medical texts, and other publications.^12^ Such an approach is necessary since evidence for specific accounts of corporeal experiences of coach travel (particularly sickness) is circumstantial and patchy. It must be acknowledged that its subjects are therefore mainly wealthy middling sorts and literary elites, and often [English] men, raising obvious questions about typicality. It does not argue that negative experiences of the motion of coaches were the norm. They may indeed have been the exception. One effect of keyword searches is the risk of privileging or over-emphasising the problem. Travellers were certainly apt to stress the dangers and negative experiences of their journeys. As Sarah Goldsmith points out, endurance of physical hardships and the ‘challenges of the road’ was a bellwether of eighteenth-century masculinity.^13^ Many, perhaps even most, travellers likely regarded coach travel as routine and, unless something unusual occurred, saw little reason to record it. Presumably too, the regularity of more serious accidents in which coaches were overturned (quite apart from other issues such as highwaymen, damaged roads or bridges, etc.) would have put these lesser discomforts into proportion. References to motion and sickness might therefore represent the extraordinary, rather than the ordinary experience. But they do reveal a common aspect of the embodied experience of motion and offer insights into the often-overlooked issue of discomfort in travel.

The intention is therefore to place some of the first footprints in the snow and highlight the ways in which physical discomfort (surely one of Holmberg’s ‘frailties’) can enrich our understanding of the lived experience of being in motion. The first part of the article explores the context of coach travel. It then turns to debates about the supposed effects of different types of motion upon the body, before finally exploring embodied mobility and the somatic experiences of coach travel.

## ‘The motion of the coach’

Coach travel increased markedly in early modern Britain. After 1650, the introduction of stagecoaches, more frequent and structured journeys, improvements in road infrastructure—notably Turnpike roads—and developments in the design and technology of coaches all served to facilitate longer and comparatively easier travel by road. According to a study of the number of towns served by regular coaches in England and Wales in the seventeenth and eighteenth century, the number of public services grew by 36.5% between 1637 and 1681 and again by 64% between 1681 and 1715.^14^

Nevertheless, travel by coach could also be slow and uncomfortable. In the century after 1680, most coach journeys were short, and generally between 1 and 60 miles.^15^ Average speeds were slowly increasing, but from a low base: before 1750 average speeds of public coaches from London have been estimated to be less than four miles per hour.^16^ Journeys were often broken for various reasons, including changing horses, running repairs, short or overnight stops at inns, bad weather, or impassable roads. In 1667 the diarist Antony à Wood took two days to travel to Oxford from London by coach, and others noted a similar duration at the end of the seventeenth century. Longer journeys from London to towns such as Exeter, Nottingham, York, and Coventry could take anything from four to six days depending on conditions and involved multiple changes of coachman.^17^

The sensory experience of the journey varied widely. As Ben Jackson has noted, coach travel was an ‘actant assemblage’ of different factors, human, animal and material.^18^ Much also depended on the type of carriage, and whether it was privately owned or a public service. Private carriages, for the sole use of owners, their families and friends, were an expensive luxury. Since they employed the driver, owners had control over journey, route, destination, and speed, and could curtail or extend it as they pleased. For the wealthy, in their private carriages, the combination of speed and motion—along with the materiality of the coach and its fittings, could be exhilarating, as well as a satisfying reminder of their own wealth and status.^19^ The cheaper alternative, and one taken by lower gentry, middling sorts, and below was to take one of the scheduled public coaches, such as the mail coach or the growing numbers of regularly advertised ‘flying machines’. Given the general paucity of records specifically detailing discomfort, it is often difficult to discern which type of coach is being used: where it is clear from a source this will be indicated. But the strong suggestion is that public coaches were more often the cause of complaints.

The healthiness of coach travel was debated in the eighteenth century, and strong arguments were advanced on both sides. As will be shown, the idea that it was a form of exercise, and therefore beneficial, appeared in various sources. In the early modern period motion was central to the maintenance of health. Movement was a key tenet of humoral medicine, preventing the harmful pooling of corrupted humours that threatened sedentary bodies. The type of exercise was important though: not too vigorous for fear of enervating or overheating the body but instead regular and moderate. The vigorous motion of a coach, although less preferable to gentler movements such as walking, was still valuable.^20^ Rather than deliberate, self-conscious movements, riding in a coach required constant adjustments to bodily position, tensing and bracing and sometimes even holding the body’s weight entirely, as the passenger clung on to straps over bumpy roads, to maintain balance or stay on their seat.

Some argued that the dynamic motion of a coach journey could be invigorating and even potentially useful in treating some medical conditions.^21^ Sceptical of the supposed successes of the Irish faith healer Valentine Greatrakes in curing various disorders by touch, for example, Reverend John Beale wrote to a friend that he believed that at least one recent ‘cure’ more likely resulted from the ‘jogging of the coach’ than to Greatrakes’ healing hands.^22^ In his 1695 treatise on ‘Chronical Diseases’, reflecting on his own experiences, John Pechey regarded coach travel as exercise which, ‘for consumptive people does sometimes a great deal of good’. But caution was also needed. Like any form of exercise, the body needed gradual exposure to build tolerance and gain the most benefit.^23^ Eighteenth-century medical texts too still regularly discussed the anodyne effects of coach travel. In some cases, notably gout, jaundice, bladder, or kidney stones, the motion of the coach was viewed as beneficial.^24^ Whilst these were theoretical discussions, not meant for a popular audience, coach travel clearly raised new questions about the effects of motion on the body. In 1757 the physician Thomas Coe argued that the shaking of the viscera and abdomen elicited by coach travel might loosen the ‘biliary concretions’ that caused stones, bringing relief to the sufferer.^25^ Over good roads the effect of a stagecoach journey could be pleasant and even soporific. Advocating the benefits of ‘gymnastick exercise, Francis Fuller suggested that the motion of the coach ‘more or less dispose[s] all people to sleep’. The faster the coach went, he added, the more likely was the passenger to doze.^26^

Indeed, arguments amongst medical practitioners for the potential health benefits of coach travel persisted into the early nineteenth century. Although the influence of humoralism was receding by that point, ideas about the supposed health benefits of coach journeys remained remarkably consistent. Whilst all exercise was potentially conducive to health, that which was too vigorous, resulting in the ‘expenditure of power’ might debilitate or exhaust the system. Forms of movement that challenged individual body parts and muscles were considered a superior alternative and, for some, travel by carriage was the best means of achieving it. Key to this was the ‘passive or oscillatory motion’ communicated to the body by the carriage as it travelled. Constantly compensating for the movement ‘required the action of many muscles’.^27^ While ocean travel offered healthful fresh air, the lurching, horizontal, and vertical swings of a ship were less salutary. Coaches such as the ‘Berlin’, with long springs, could cause similar swinging motions and were therefore less preferable than the common stage, which had shorter springs and more rapid and vibratory motion.^28^

For others, however, coach travel was not only unhealthy but harmful. The often-poor-quality ride was commonly described by travellers and lampooned by satirists. In 1702 a character in Edward Ward’s *London Spy* described both the noise of his coach as it rattled over London roads, and the sensation for passengers ‘jumbled about like so many pease in a child’s rattle’, arriving with bruised elbows and shoulders due to the ‘thumps and tosses of our ill-conceiv’d engine’.^29^ For some, the pain brought on by the jolting movement could make coach travel unsupportable. Sufferers of bladder and kidney stones would likely find that travelling by coach caused pain and tenderness and might even induce urination.^30^ Discussions of coach motion were often couched in the terminology of violence. Some dictionaries indeed defined the word ‘jolt’ specifically in relation to coach travel. In Dyche and Pardon’s 1748 *New General English Dictionary*, for example, ‘jolt’ referred to ‘a violent shaking occasion’d by the irregular motion of a coach or wagon’.^31^ The use of the word ‘irregular’ here highlights another important point: coach travel was not a uniform motion. It was unpredictable and irregular, challenging passengers’ bodies and senses. Indeed, the precise nature and effects of this motion raised new questions in popular publications. In 1726 an edition of the *British Apollo* included two thousand ‘curious questions in most arts and sciences’, some sent in by readers. One contributor asked why, when sitting in a coach with eyes shut, ‘the coach seems to move the contrary way to what it actually does’.^32^ The answer provided was that, without the horizon as a reference point, ‘animal spirits’ floating in the brain produced a false sense of direction.

Whether or not travelling by coach would help, heal, or harm, therefore, was a matter of debate across the period, as amidst broader discussions about the supposed effects of motion upon the human body. It is to those debates that the next part of the article turns.

## Embodying coach travel

If the idea that motion of various kinds might cause physical and mental discomfort was apparently in common currency, how was this understood and experienced by individuals? To fully recover the somatic experience of premodern coach travel it is necessary to understand the sensory barrage that it entailed. The poem noted at the beginning of this article offers a useful framework through which to compare the experiences of actual travellers.

### Comfort and space

As John Crowley has argued, modern ideas about physical comfort date only to the late eighteenth century. Before this, ‘comfort’ generally signified moral, spiritual, and emotional matters, while ‘discomfort’ applied to emotional rather than physical unease.^33^ Crowley’s work highlights the dangers of back projecting modern frameworks of comfort onto premodern actors, but eighteenth- and nineteenth-century travellers certainly experienced physical discomfort. As discussed below, everything from the type of motion to the environment and the coach challenged and afflicted the bodies—and minds—of passengers. ‘Discomfort’ is here used in its modern sense, considering that what is often being described in sources are physical symptoms and sensations. Added to this were the classed and gendered dimensions that further shaped the experience. Sharing the confined space of the coach raised various issues, as the above examples suggest, leading to problematic assumptions and awkward encounters. Journeys were not experienced by the same people in the same ways, and neither was the prospect of close physical proximity.

The interior space of the coach was often neither easy nor comfortable. ‘My journey was not so bad as usual in a stage coach’ wrote the poet Thomas Gray to his friend Dr Thomas Warton’ in October 1753. This was damning with faint praise. The journey was tolerable…but by no means typical.^34^ The reasons for this were manifold. Sociability, for example, underpinned public life in the eighteenth century and crafted conversation was a key element of polite performance. But coach travel threw together individuals of different temperaments, backgrounds, and status, exposing passengers to opinions, values, and morals potentially inimical to their own. In the street, park, or even social events, encounters with others could be carefully curated and terminated once no longer useful or pleasant. Inside the coach, however, there was nowhere to hide.

As Daniel Jutte has argued, coach travel gave rise to ‘unmediated bodily proximity’ between social classes.^35^ While travellers might display curiosity towards people of different national backgrounds, those of a different class were likely to be treated with disdain.^36^ The manners, behaviours and habits of other passengers could indeed prove discomfiting. Writing to his brother, the Irish playwright and barrister Arthur Murphy complained of the two ‘wrongheads’ with whom he was forced to share a long journey, one of whom constantly either took, or talked about, medicines. Murphy described himself as ‘benumbed’ by the experience and wished the code of conduct for coaches suggested by the Spectator in 1712 was brought into law.^37^ He was referring to a letter from ‘Rebecca Ridinghood’ which complained about the loose manners and rough conversation of travellers, especially when in the company of women, and asked that such a document be pasted on the walls of every coach.^38^ The rough and readiness and jarring conversation of fellow passengers was a constant source of consternation.^39^ Annoying personal habits could be worse. In 1777 the utilitarian ideas of philosopher Jeremy Bentham were challenged when he endured hours jumbled and squashed into a coach reeking of tobacco, with a soldier, a judge, and a Welsh drover, who smoked, snuffed, or chewed it constantly.^40^ Bentham’s individual happiness was clearly affected by the loud snuffings and hawkings, alongside the smell and debris of the tobacco. But, as an educated man from a wealthy background, given that two of the men were of lower status, it seems likely that class and assumptions about morality also fed his distaste.

Space inside the coach was indeed at a premium, leading to physical and psychological discomfort, often couched in terms of ‘squeezing’. Writing to his father in 1656, the English philosopher John Locke complained of enduring a ‘thousand squeezes’ in a three-day coach journey packed in tightly with six passengers. He was also physically repelled, to the point of nausea, by the corpulent body of one woman.^41^ A century later, ‘Mr Wilson’, packed tightly into a coach with the corpulent philosopher David Hume found the jarring of the coach doubly problematic since every ‘jolt we receiv’d’ threw the ‘enormous weight [of Hume] onto his little carcass’, at which Wilson ‘complain’d grievously’.^42^ Being crammed into tight spaces for long periods also had other unfortunate effects. Twice in his diary, Samuel Pepys recorded pain in his ‘cods’, specifically caused by compression of his testicles through being ‘being squeezed’ into a coach.^43^

If issues were raised by the mixing of social classes, gendered encounters added further layers of complexity linked to propriety. In 1706 ‘jumbled’ together with his manservant and four women, described as ‘damn’d battered jades’, one of whom was sick and ‘useless’, the playwright William Wycherley was concerned for his moral as well as physical fortitude. Writing to Alexander Pope he claimed to be so suspicious of the lascivious nature of his fellow passengers that he feared a ‘fall’ inside the coach with the women more than he did one out of it.^44^ Wycherley’s fears reflect wider concerns about the potential for the enclosed privacy of the coach interior, and indeed its motion, to encourage illicit sexual behaviour. As Charlotte Campton has argued, eighteenth-century literature and satires emphasised the bawdiness of the coach interior.^45^ Aside from morality, men complained about the incessant chatter of female passengers. For women, the shared space and close physical proximity of the coach raised issues, especially if they travelled alone, which generally required permission from husbands or fathers.^46^ On the one hand it was argued that coaches offered women more freedom and independence, were relatively safe and less exhausting than other forms of travel. On the other, (albeit in the eyes of male writers) they were set up as risky spaces for women, where the unwonted attentions or salty conversation of fellow male passengers could corrupt feminine sensibilities.^47^ As Yoojung Choi suggests, the experiences of Delariver Manley in fact lean towards the former. Manley’s fellow passengers included several single women, travelling alone, and from across the social scale, suggesting ‘self-education and independence’. She did, however, remark on the lack of manners and decorum amongst male passengers.^48^

If space was a problem, there were constant complaints about the heady smells and fuggy atmosphere caused by the bodies of fellow passengers. At best this was unpleasant; at worst nauseating and even unhealthy. Beliefs in the harmful effects of ‘miasma’ were commonplace in premodern medicine. Good and bad air, along with moderate temperature, were key determinants of health, and such beliefs lingered long into the eighteenth century.^49^ In 1686, Robert Boyle noted that ‘Over-charging of the Air [inside the coach] with the fuliginous Reeks of Men’s Bodies’ could see passengers subject to ‘Faintness, or cast into a Swoon, by the closeness of the Place’.^50^ Decades later, the ‘reeking scent’ of fellow passengers led to the practice of carrying scented handkerchiefs, and even well into the nineteenth century things had changed little.^51^ But eighteenth-century passengers complained constantly about everything from the ‘nasty scents’ of children to the noisome ‘tributes to nature’ that emitted from the backsides of rich and poor alike.^52^ Amidst nineteenth-century obsessions with ventilation and clean air, anyone ‘fresh from the morning air who enter[ed] a coach in which several persons had been close stewed during a long night’ risked colds and fevers.^53^

The metaphor of ‘stewing’ was not uncommon. In 1803, Jeremy Bentham again picked up his pen to deplore the ‘stewing boxes they call coaches’.^54^ Weather, and season exacerbated the problem. In cold weather passengers shivered and were perhaps even glad of the proximity of others for warmth. Hot, muggy conditions, however, curdled the already heady atmosphere of the coach. Recalling a journey to Chester, poet Alexander Pope, a frequent coach traveller, ‘felt the extream heat of the weather, the inns, the roads, the confinement and closeness of the uneasy coach’.^55^ It is interesting to note the use of the term ‘uneasy’, suggesting both physical and mental disquiet. In a coach from Bath in 1798, packed tightly with five passengers, including a corpulent woman who ‘half filled it’ John Dickenson found the air inside the coach to be ‘intensely tart’.^56^ To combat the problem, prospective travellers were advised to sit next to a window and ideally keep it at least partially open. The 1793 *Useful Instructions for Travellers* particularly stressed the need to open carriage windows frequently to refresh the air.^57^ It is indeed interesting to note that references to the lack of fresh air in the coach, and its effects occur—at least in this sample—more frequently amongst women. For Elizabeth Montagu, heat, the ‘motion of the coach’ and a fellow passenger’s chatter had left her ‘head so giddy I hardly know what to do.^58^ I another letter to her sister, Montagu suggested that ‘if your nerves, your spirits and your stomach are all out of order’, she should travel in an open, one-horse vehicle, which Montagu herself had found salutary.^59^ In 1785 Jenny Brownsword ‘had a very unpleasant journey being very sick all the way’ in a coach travelling through Derbyshire. Her solution was to ‘ask the postillion to drive quick’ as well as opening all the windows.^60^ Whilst obviously not limited to women, references to ‘giddiness’ appear far less in male descriptions. The reference to perhaps the signal female malady of the eighteenth century—nerves—is also interesting here, suggesting again that susceptibility to motion might vary in nuanced ways according to gender.

### Experiencing motion

Once the traveller had become accustomed to the physical and psychological effects of the tight space, the effects of motion had to be negotiated. Some spoke in favourable terms about the positive, even restorative, effects of a coach journey. In a letter to his cousin, the Scottish-Canadian explorer and fur trader Alexander Mackenzie was suffering from a ‘disagreeable and most uncomfortable disorder’. Whilst walking brought on ‘headache, stupor or dead pain’, listlessness and apathy, ‘exercise in a carriage, if not violent, has a beneficial effect. The great Doctor Hamilton of Edinburg[h] calls it a shake of the constitution and I am acting now under his guidance’.^61^ Here, though, the phrase ‘if not violent’ is telling, and the contrary idea, that the violent jolting motion of a coach could upset the body, was also common in medical debates. In 1721 it was reported that, after suffering for many years from pain and jaundice caused by bladder or kidney stones, the ‘motion of the coach’ on a particular journey caused a man to void a stone, together with ‘choler and gravelly matter’.^62^ These ideas were not restricted to medical practitioners, since both male and female travellers reported themselves ‘unable to bear’ the motion of the coach. Samuel Bentham asked his son Jeremy how he would possibly countenance a sea voyage, ‘you who can scarcely bear the motion of a coach’.^63^ In 1816 Hester Thrale Piozzi, noted being ‘in no wise [able] to bear the motion of a carriage’, and elsewhere that ‘poor dear Miss Williams’ was rendered housebound by ‘her incapacity to bear the motion of a carriage’.^64^ Given that she was an inveterate coach traveller, both in Britain and across Europe, this suggests an unusual occurrence.

When combined with the often-violent movements of the coach, too, the tight space provided multiple opportunities for injury as bodies tumbled into one another or barged against the walls and windows of the vehicle. The word ‘jolting’, noted in dictionary definitions of coach motion above, was also used negatively by passengers. Following an accident in which his coach overturned, and his companions were injured, Jeremy Bentham noted that the condition of one man a few days later was ‘so bad as to render the jolting of a carriage dangerous’.^65^ Albeit commenting here on luxurious private coaches, rather than public, George Cruikshank’s 1797 ‘Symptoms of Jolting’ (fig. 1 below), from a book satirising high and low born English stereotypes, provided four different takes on the dangers of coach travel, from the physical dangers of clashing heads or falling off seats, to the injury to pride and status of wigs flying off. In each case, the passengers’ confinement is emphasised by their positions within the coach and also the tightly framed images. Its companion, ‘Jolting Preventatives’, (fig. 2, below) suggested how such dangers might be avoided by holding straps, paying attention to posture and sitting positions.^66^ In public coaches, the lack of space must have been more keenly felt.

**Figure 1.**
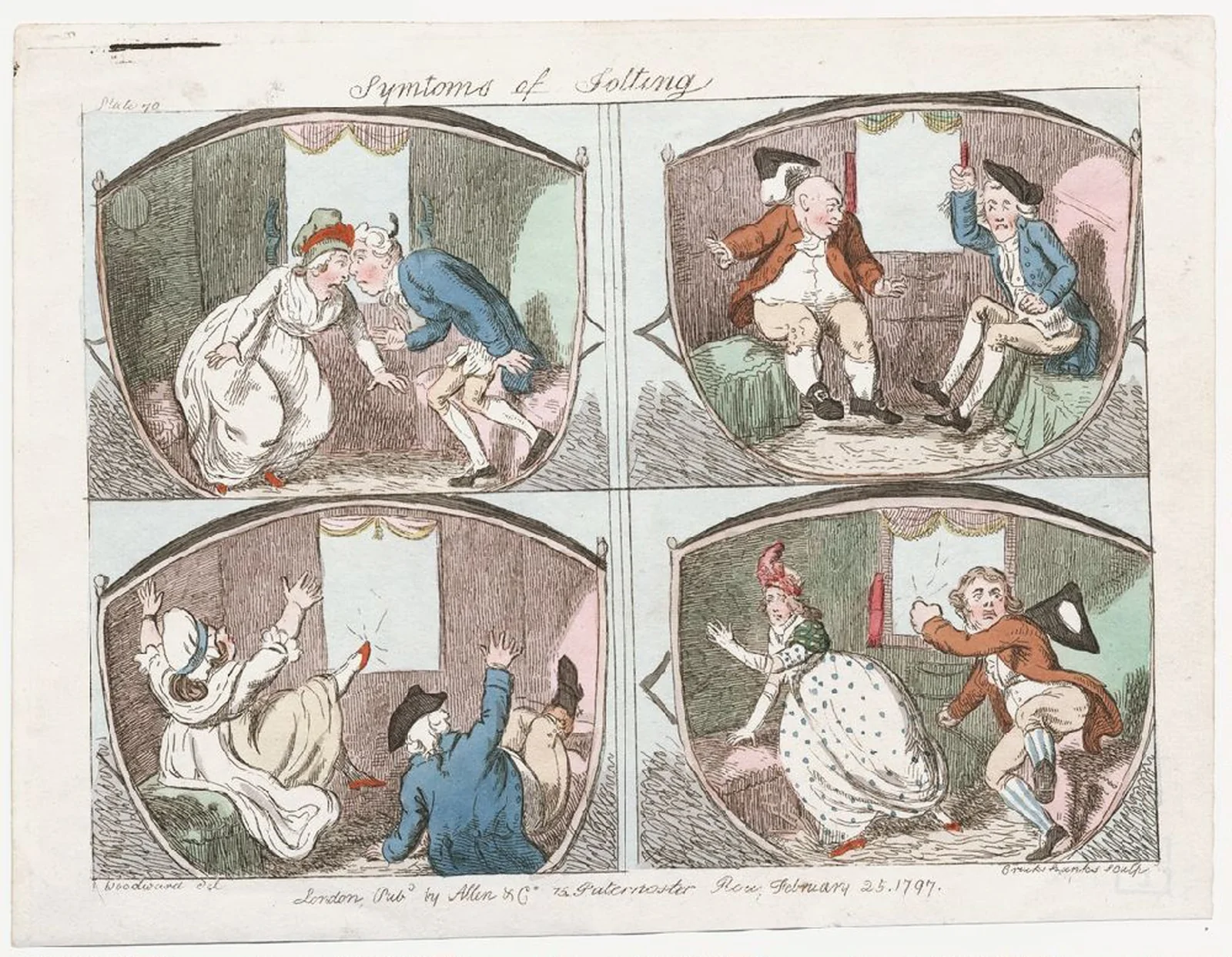
Symptoms of jolting, George Woodward, *Eccentric Excursions or Literary and Pictorial Sketches of Countenance, Character and Country in Different Parts of England and South Wales* (London: Allen and Co., 1796), 164. Image Courtesty of the Lewis Walpole Library, Yale University.

**Figure 2.**
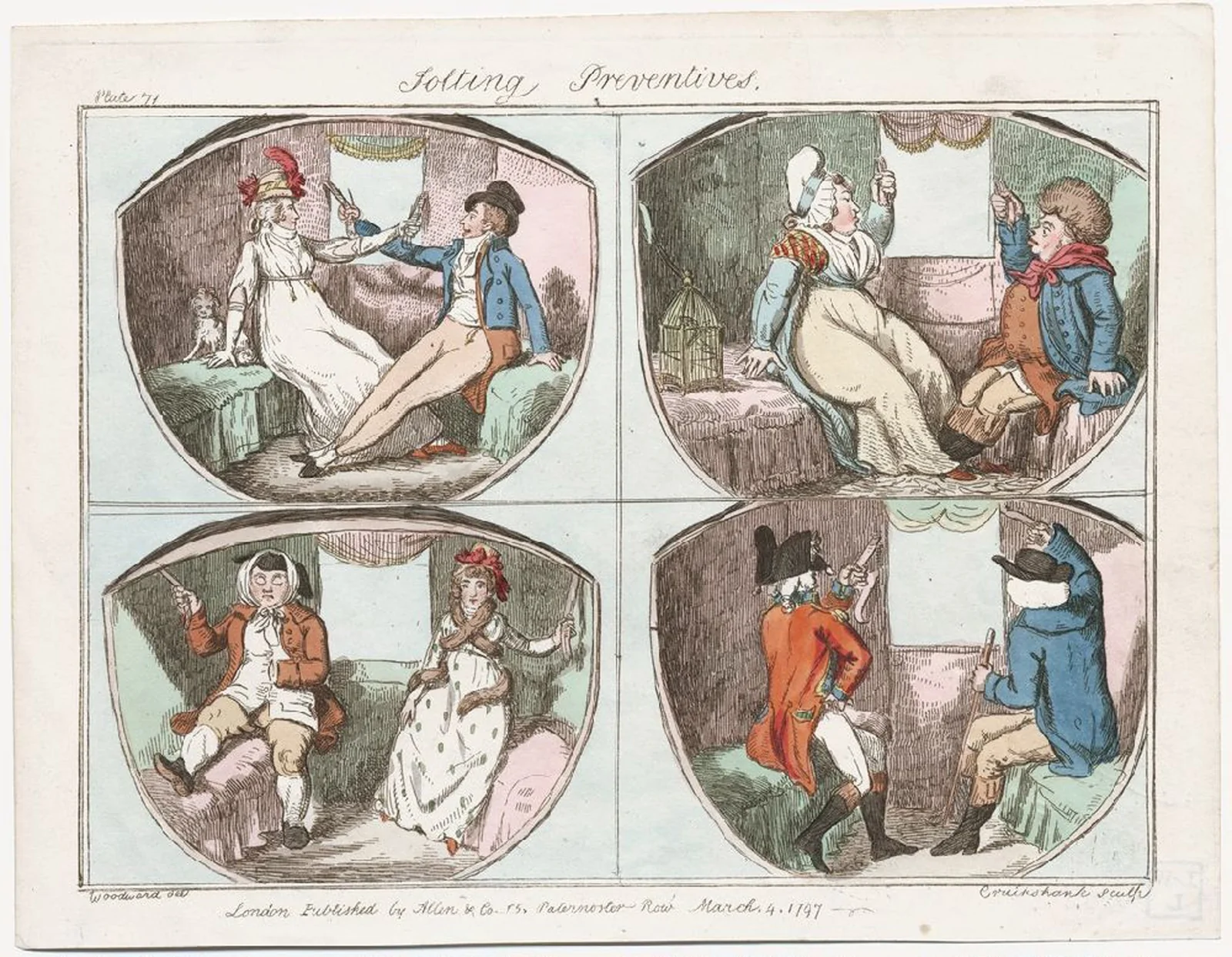
Jolting preventatives, George Woodward, *Eccentric Excursions or Literary and Pictorial Sketches of Countenance, Character and Country in Different Parts of England and South Wales* (London: Allen and Co., 1796), 166. Image Courtesy of the Lewis Walpole Library, Yale University.

Evidence shows that jolting often combined with other sensations to disturb both physical and mental wellbeing. Again, this was reported by both female and male passengers in similar terms. Travelling through South Wales in 1809 Sophia Hoare was ‘heartily tired with the jumbling and jolting of our miserable vehicle’. On another occasion she was ‘jumbled and jolted [by the coach] till all my bones ached’.^67^ In 1822 Anne Lister was ‘exceedingly jolted’ and on another occasion ‘shockingly jolted’ by the movement of her coach across bad roads.^68^ In a letter to his friend, the barrister Samuel Rose, William Cowper deplored the ‘many severe shocks and jumblings’ of his coach in a journey over the Sussex hills.^69^ Recounting the same journey to another correspondent a few days later its effects lingered, though the language was markedly different: ‘After three days’ confinement in a coach and suffering as we went all that could be suffer'd from excessive heat and dust’.^70^ For many, then, the violent movement of the coach rendered it something to be ‘suffer’d’ rather than enjoyed. Cowper also suggested that ‘outside’ passengers, despite being exposed to weather, might have suffered less from uncomfortable motion since the top of the coach was ‘farthest removed from the point of joltation’.^71^ Recalling William Wycherley’s early fears for his morality, it is worth noting too that, in early modern literature, the term ‘jolting’, noted in the poem at the start of this article, and by other sources throughout to describe the distinct movement of the carriage, also stood as a shorthand for sexual acts.^72^ There is insufficient evidence to demonstrate conclusively that this connotation was understood, accepted or implied by travellers in the context of their narratives, but the close connection between the jolting motion and the sexual act is nonetheless interesting.

For impaired passengers, the challenges of both the motion and the journey by coach were made more acute. The English poet and satirist Alexander Pope regularly bemoaned the turbulence and discomfort of the coach. Pope suffered from various health conditions, including tuberculosis, skeletal deformity, and stunted growth, which left him in constant pain, and doubtless exacerbated his dislike of travel. Writing to the author Joseph Spence he began by hoping that ‘this finds you in repair after the concussion of the stage coach’.^73^ In another the prospect of a coach journey ‘so much tumbling *alone* in my present circumstance’ led him to decline an invitation to visit Bath unless he could find others to accompany him.^74^ As his friend the surgeon William Cheselden told him, ‘a coach would be a herse (sic) to me if I went in one alone’.^75^ His dislike of coaches extended to his poetry. In ‘The Rape of the Lock’, coaches were used as symbols of the superficiality and ‘varying vanities’ of the characters.^76^ Jeremy Bentham feared that his ‘malady’—gout and bladder stones—was irritated by ‘motion in a carriage’ and he ‘feared the consequences of such a journey’.^77^

Jolting was enough of an issue to concern coach manufacturers. As Ben Jackson has shown, eighteenth-century coach design was a site of continuing innovation, involving experimentation with different materials and devices to try and mitigate the physical challenges and effects of the journey.^78^ The operators of coach services were certainly at pains to stress their consideration for the physical comfort of their passengers, often stressing their use of new innovations or technologies to mitigate the effects of motion. Edmond Hammond’s ‘Elegant Flying Stage Coaches’, were contrived in a ‘most easy and comfortable manner for the satisfaction and expedition of passengers, being hung upon four steel springs’.^79^ In his essay on carriage construction, Thomas Fuller argued that springs placed in the axles of coaches prevented uncomfortable shaking and increased the ‘ease’ of passengers.^80^ The language of ease and comfort is notable. Manufacturers and patentees similarly talked up the qualities of their inventions in offsetting motion, and increasing comfort. In 1830, Thomas Brown of Birmingham patented a coach ‘of a lighter kind’, which incorporated four springs running horizontally along the side of the coach, ‘to obviate much of the jolting motion experienced by passengers’.^81^ The extent to which specific changes improved the experience for passengers is harder to discern.

## Motion sickness

Amongst the c. 2000 ‘curious questions’ in the *British Apollo* of 1718 was ‘why some persons travelling in a coach become so very sick?’. The answer, according to the editors, was agitation of the spirits of the brain caused by the jolting of the coach, in turn causing retardation of blood circulation.^82^ Despite the abundance of travel narratives, little work has yet sought to historicise this uncomfortable malady. As such we know little about how it was understood or experienced. There are potential issues, however, not least the dangers of mapping modern ideas and fears about ‘motion sickness’ on to the past. It is not clear, for example, how far, or even whether, motion-induced vomiting was understood as a specific ‘condition’ or even a problem or deterrent to travellers in this period. Although it was presumably physically uncomfortable and unpleasant, premodern people were used to vomiting. It was actively encouraged in treatment regimens and even as a routine ‘spring clean’.

Matters are further complicated by terminology. Travellers clearly recognised that motion could cause nausea or vomiting, but the terms used, and their specific meanings, lack fixity. According to the OED, for example, the terms ‘motion sickn(ess)’ and ‘travel sick(ness)’ as blanket terms for motion-induced vomiting did not exist until the late nineteenth century. The earliest terms for sickness caused by a specific form of travel related to maritime travel. The term ‘seasick’, for example, as a bodily state or sensation, dates to the mid-1500s, whilst ‘seasickness’ and/or ‘sea distemper’ as specific conditions, emerged in the early seventeenth century. But around 1700, the term ‘coach-sick’ began to appear as a specific form of malady.^83^ ‘Carriage sick’ was also used but less frequently.^84^ This raises questions about whether the modern concept of ‘motion sickness’ as a blanket term was even understood by contemporaries to be anything like the modern interpretation or was instead situational according to the type of transport. In fact, sickness caused by motion occupied an ambiguous position within medical theory. Whilst vomiting was medicalised, being both a symptom and a treatment, sickness as an *effect of motion* itself was potentially not. It is worth briefly exploring early modern debates about the causes of vomiting and the supposed effects of motion upon the body.

In the early modern period, as it had been since antiquity, vomiting was a key form of treatment, as well as a symptom of illness. Emetics were part of the suite of evacuative treatments through which corrupted humours or foul substances were violently driven from the body, restoring vital balance. The potential for motion to cause vomiting has been recognised globally since antiquity, often construed as a failure of the body to adapt to types of motion, and notably waves.^85^ Whilst Greek and Roman physicians blamed sea travel, elsewhere there was recognition that different types of transport, with their own characteristics of motion, might also be problematic. In ancient Chinese medical thought, for example, sickness was recognised to occur in different types of transport, including land based. Here, for example, children were regarded as particularly vulnerable to motion-induced sickness, which was observed to occur when travelling by carts or litters as well as by sea.^86^ Discussions of the effects of motion could often be found within debates about the causes of vomiting, which were themselves generally split into distinct categories. Where vomiting occurred as a symptom of disease—either alone or with others—of a particular condition it was regarded as ‘natural’, enabling ‘Nature to separate and squeeze out those humours which are incompatible with and praeternatural to it’.^87^ Alternatively, vomiting might be ‘artificial’, caused by external factors that served to upset bodily equilibrium. These could include deliberate taking of emetic substances as part of a treatment regimen, surfeits caused by over-eating or drinking, or excessive or sudden movement. In his 1685 treatise on vomiting, Hugh Chamberlain cited ‘giddiness’ and ‘sea-voyages’ as two examples of movement-induced sickness.^88^ Lazare Rivière listed 17 causes of ‘artificial vomiting’, one of which was ‘sayling at sea especially in a southerly wind’.^89^

As Ulinka Rublack has noted, there was certainly a growing sense that motion, and particularly violent jolting of the body such as that caused by travelling in a coach over rutted roads, could upset bodily balance.^90^ Edward Strother’s *Practical Physician for Travellers, Whether by Sea or Land* noted that ‘tender and sickly’ people might be susceptible to the unsettling experience of ‘excessive motion’.^91^ For Strother, vomiting was caused when violent motion, whether aboard ship or on land, caused the nerves to act ‘too forcibly on the stomach’.^92^ In his reading, vomiting resulted from an unusual motion hitherto not experienced by the individual.^93^

If physicians generally accepted that motion of various kinds could cause vomiting, however, they could not agree on which organ was either most affected or responsible. One survey of ‘natural knowledge’ in 1665, for example, argued that nausea occurred when the body was subject to violent or sudden external motion ‘to which it contributes not itself’—in other words involuntary. This was due to the sudden agitation of spirits which in turn acted upon the humours to cause vomiting.^94^ Noting that ‘some being in a ship or riding on a coach are troubled with huge Vomiting’, Thomas Willis argued that the problem lay not with agitated humours but rather the stirring of animal spirits in the brain that stimulated ‘emetick contractions’.^95^ Others stated instead that the action of motion upon the stomach was the predominant cause of nausea. Michael Etmuller’s chapter ‘of Vomiting and Loathing Meat’ regarded vomiting as occurring only when the ‘irritating cause is lodg’d within the stomach’.^96^ In his *Mysteries of Opium Reveal’d* (1700), John Jones agreed, argued that one of the chief causes of vomiting was ‘swimmings in the head’. Rather than, as he suggested was widely believed, the brain being first affected by the sensation of motion, Jones argued that it was the stomach that was ‘first offended by the motion of the coach or ship’, which, ‘by reason of its most exquisite sensation’ then caused the ‘swimmings’ characteristic of motion sickness.^97^

There were also alternative arguments: one was that the motion of a ship at sea could not be considered a primary cause of sickness since violent movements were equally replicable upon land without the same effects.^98^ As such the action of the sea air, rather than the motion of the waves, was blamed. Another blamed the ‘equality of motion’, rather than jolting or sudden movements, especially for ‘persons unus’d to go in a coach [who] vomit sooner when the Coach goes in a smooth and even field than upon rough ways’.^99^ The lack of specific references make it hard to gauge the effects of different types of coaches, but it seems reasonable to assume that material construction and suspension might play a part. and even perhaps whether the coach was enclosed or, as with a gig, completely open. It would be interesting to know, for example, whether the experience of modern sufferers of car sickness who are not affected when they are driving the vehicle existed in the past, for example in the case of phaetons, which were self-driven.^100^

The later eighteenth century, however, did bring about some changes in ideas about the healthiness of motion, complicating ideas about how, why and indeed whether it caused vomiting. The idea, noted above, that motion, especially over uneven surfaces was a form of exercise, was still in common currency. All kinds of motion challenged the ‘calorique’ of the body, disturbing its equilibrium and requiring it to compensate. When this occurred gently, or in regular or accustomed motion, it could be invigorating.^101^ Motion became problematic, however, when it involved dramatic ‘swings’ or sudden or violent changes such as lifts or drops. For the physician William Okely, the offending movement in coach (and sea) sickness was likened to the ‘peculiar, indescribable sensation’ of being on a swing as it rapidly dropped from the top of the arc. Equally, however, just as with seasickness, it was assumed that once the initial squeamishness abated, the body would soon acclimatise to the motion of the coach, and no further problems would occur.^102^

While the precise effects of motion were debateable, then, its capacity to cause vomiting was widely accepted. It is noteworthy that sea travel and land travel were generally discussed together, suggesting that they were regarded as part of the same causal framework. Equally, however, the fact that they were separately identified highlights recognition of the differing types of motion. On sea, as some noted, problems could arise either through the vertical motion of the ship as it crested heavy waves, or the linear heaving and swaying through seas abeam. On land, the constant vibration of the coach, or the jarring crashes and bangs over rough roads were the chief causes of nausea. In either case, the causal factor was the action of involuntary motion.

Evidence for the coach sickness of individuals is patchy, but points to the misery of the experience for both men and women. In 1767 Jeremy Bentham arrived late at his friend’s house ‘after a very sickly passage as far as Copdock’.^103^ The term suggests that Bentham and perhaps his fellow passengers were nauseous or sick throughout, evoking the discomfort of the journey. The English parson-naturalist and author Gilbert White in 1769 wrote, in response to an invitation to travel from his home in Hampshire to his friend’s house in north Wales, ‘I have neither time nor bodily abilities adequate to so long a journey. And if I had time I am subject to such horrible coach-sickness, that I should be near dead long before I got to Chester’.^104^ This quote again highlights the deeply embodied experience of coach journeys. Coach travel called on various ‘bodily abilities’. The vomiting it elicited was not just uncomfortable but ‘horrible’. Keeping in mind the epistolary conventions of the ‘heroic sufferer’, the choice of the term ‘near dead’ is still striking.

Other factors could exacerbate problems for queasy travellers. Facing backwards, that is, against the direction of travel, could bring on motion sickness. This was certainly a recognised issue and a subject of some philosophical debate. Robert Boyle noted in 1683 that passengers ‘riding backwards in a Coach are not only much distemper'd in their Heads, but are made very sick in their Stomachs, and forced to Vomit, as violently and frequently, as if they had taken an Emetick’. This ‘distemper’, he believed, would quickly abate once the person left the coach.^105^ More than a century later, William Okely suggested that the reason that people often experienced coach sickness when facing backwards was simply because the human body was naturally accustomed, as in walking, to moving forwards.^106^ Evidence from individual travellers confirms that the experience could also be an ordeal for the sufferer and fellow passengers alike. In a letter in 1765, ‘Miss Talbot’ noted being glad that her travelling friend was not trapped in a ‘snail-paced stage coach, with two tars, one smoaker [and] two fat gentlewomen (one of them sick with riding backwards)’.^57^ Here, also, class may have affected the experience further. A (possibly imaginary) letter from a female servant to *The World* in 1753 noted that her employer forced her to sit backwards when travelling by coach, even though she was often sick—something she attributed to being ‘not much used to a coach’.^107^

This last point is interesting, suggesting that unfamiliarity or inexperience with mechanical travel could itself be a cause of nausea, but one that might improve once an individual was ‘used’ to it. In the seventeenth century there were suggestions that simply being unused to the motion of vehicular travel by coach or wagon might be enough to cause a ‘bad stomach and restlessness’ or just a general sense of discomfort.^108^ The eighteenth century saw a specific term for this: In Samuel Johnson’s dictionary, to be ‘UNWAYED’ was to be unused to travel.^109^ The ‘motion of the coach’ was one of the ‘considerable hardships’ noted by John Griffith in a journey from London to Chester in 1779.^110^ In a letter to a companion, the English merchant and philanthropist Jonas Hanway noted that ‘the motion of the coach did not agree with me’ but that he was ‘*restored to himself*’ [my emphasis] soon after alighting, suggesting that he felt almost disembodied by the experience.^111^

## Conclusion

This article has sought to reveal the complex sets of ideas that revolved around the body and motion in the long eighteenth century, including the various supposed effects of land-based travel, and particularly factors such as corporeal discomfort and motion-induced sickness. As it has argued, eighteenth-century coach travellers were placed into new circumstances and conditions which they were not necessarily familiar with or adapted to. Motion of various kinds was understood to cause nausea and vomiting but, as debates in medical texts show, precisely how this occurred, and which parts of the body were responsible, was far from clear. Whilst we cannot know the proportion of individuals affected, or how typical their experience was, there is enough evidence to suggest that it was not uncommon. More broadly, the article highlights the significance of motion and transport in shaping embodied and materially engaged experiences. By turning attention to the *dis*comforts associated with travel, it offers new opportunities for expanding the work of scholars such as Karen Harvey on embodiment as lived experience. At the same time, it contributes to the emerging literature on embodied mobility by introducing a fresh dimension—what might be described as kinaesthetic or dynamic embodiment.

As has been shown, for example, for many people, the experience of motion, as well as the type and environment of the vehicle, was both physically uncomfortable, and unsettling. Coach travel combined many corporeal and sensory experiences. First was exposure to different types of movement, voluntary and involuntary. Nominally, coaches—and those within them—moved uniformly forward. But how this was experienced depended greatly on factors including speed, road type and state, the type of the coach and its technology. Motion could mark the material body in various ways, causing bumps and bruises or sore limbs. At worst bodies might literally be thrown together, adding moral and sexual elements to physical discomfort and pain.

While the somatic experience of early rail travel has attracted attention the limited study of sources here suggests much potential in looking further back to the material entanglements between passengers and the vehicles they travelled in. Seated within (or in the case of ‘outside passengers’, on) the moving coach, bodies and machines were closely bound. Backsides sat on seats that might be plush and padded, or hard and uncomfortable. Hands touched wood and leather fittings, cloth curtains, tightly held on to straps, and perhaps wiped the condensation from cold windows that obscured their view. Motion involved both the interior and exterior of the body, and in the case of motion sickness uncomfortably combined the two.

As the narratives and letters of past travellers reveal, various aspects of coach travel served to cause both physical and psychological unease. The atmosphere, heat or cold, and smell of the coach strongly affected the embodied experience. Squashed, stinking and ‘stewed’ bodies nauseated other passengers, combining or adding to the already complicated experience of motion. The intense olfactory ‘soundscape’ of the coach was another factor. This cacophonous jumble of sounds included rattling wheels over rough roads, the chatter, coughing, and spluttering of fellow passengers and the clopping and whinnying of horses. Together, these elements produced an intensely sensory and embodied experience.

Evidence discussed above suggests that experiences also varied according to gender and class. Proximity to others brought into play awkward social encounters. For women, the interior of the coach brought them into physical contact with the bodies of men, raising issues of propriety. Deliberate and unwanted touching or groping by male passengers was a possibility; but perhaps more common was an early version of the modern concept of ‘manspreading’. Equally, for affluent middling sorts, sharing a coach with commoners was unsettling. Putting up with their perceived unsavoury habits was one thing, but the interior of the coach arguably blurred—or even removed—social boundaries. All passengers arrived at the same time, for example, and occupied the same space. More work is certainly needed to tease out the nuances of these encounters.

Coach travel in the long eighteenth century, therefore, was not merely a matter of conveyance but a deeply embodied encounter, one that disturbed, marked, and transformed travelling bodies. By foregrounding discomfort, sensory experience, and the material entanglements between body and machine, this study adds further weight to recent calls for more attention to be paid towards the embodiment of mobility and the effects of motion on the experience and performance of the body.

## Footnotes

1 A colloquial term for a coach, based on the common use of the phrases ‘God willing’ or ‘God Permitting’ in advertisements and timetables for coach services in the eighteenth century.

2 For examples, see Anon, ‘The Comfort of a Stage Coach’, *Bury and Norwich Post*, 13 October 1790; ‘The Comforts of the Coach’, *Hull Advertiser*, 10 August 1799, 4.

3 For a useful summary of these developments and debates see Iris Clever and Willemijn Ruberg, ‘Beyond Cultural History? The Material Turn, Praxiography, and Body History’, *Humanities*, 3 (2014), 546–66.

4 Elizabeth Craig-Atkins and Karen Harvey, ‘Introduction’, in Craig-Atkins and Harvey, eds, *The Material Body: Embodiment, History and Archaeology in Industrialising England, 1700–1850* (Manchester: Manchester University Press, 2024), 8–10.

5 For examples, see Karen Harvey and Sarah Fox, ‘Feeling Old in Eighteenth-Century Britain’, *Journal of British Studies*, 64 (2025), 1–23; Karen Harvey, ‘Men of Parts: Masculine Embodiment and the Male Leg in Eighteenth-Century England’, *Journey of British Studies*, 54 (2015), 797–821; Ben Jackson, *Material Masculinities: Men and Goods in Eighteenth-Century England* (Manchester: MUP, 2025); Sarah Goldsmith, Sheryllynne Haggerty and Karen Harvey, eds, *Letters and the Body, 1700–1830: Writing and Embodiment* (London: Routledge, 2023); Matthew McCormack, ‘Boots, Material Culture and Georgian Masculinities’, *Social History*, 42 (2017), 461–79; Joanne Begiato, ‘Between Poise and Power: Embodied Manliness in Eighteenth and Nineteenth-Century British Culture’, *Transactions of the Royal Historical Society*, 26 (2016), 125–47.

6 Charles Forsdick, ‘The Body’; in Charles Forsdick, Zoe Kinsley and Kathryn Walchester, eds, *Keywords for Travel Writing Studies: A Critical Glossary* (Cambridge: Cambridge University Press, 2019), 22–24.

7 Forsdick, ‘The Body’, 22–4.

8 Gerrit Verhoeven, ‘Wading Through the Mire: Mobility on the Grand Tour, 1585–1750’, in Paul Nelles and Roza Salzberg, eds, *Connected Mobilities in the Early Modern World* (Amsterdam: Amsterdam University Press, 2022), 3, 63–86.

9 Eva Johanna Holmberg, ‘Introduction: Renaissance and Early Modern Travel – Practice and Experience, 1500–1700’, *Renaissance Studies*, 33 (2019), 516, 522.

10 Mark Williams, ‘The Inner Lives of Early Modern Travel’, *The Historical Journal*, 62 (2019), 352.

11 Yoojung Choi, ‘“Every Jolt Will Squash Their Guts”: Women’s Stagecoach Travel in Delarivier Manley’s Letters’, *Early Modern Women*, 14 (2019), 141–51. Other focussed or peripheral studies of stagecoach travel include Roza Salzberg, *Renaissance on the Road: Migration, Mobility and Cultural Exchange* (Cambridge: CUP, 2023); Mark Brayshay, *Land Travel and Communications in Tudor and Stuart England: Achieving a Joined Up Realm* (Liverpool: Liverpool University Press, 2014).

12 The primary databases used for letters in this article are: Material Identities, Social Bodies: Embodiment in British Letters c.1680–1820. Compiled by: Karen Harvey, Helen Esfandiary, Sarah Fox, Emily Vine, University of Birmingham. Project funded by the Leverhulme Trust (2021–2025, Ref. RPG-2020-163), https://socialbodies.bham.ac.uk; *The Mary Hamilton Papers (c.1740–c.1850)*. Compiled by David Denison, Nuria Yáñez-Bouza, Tino Oudesluijs, Cassandra Ulph, Christine Wallis, Hannah Barker and Sophie Coulombeau, University of Manchester, 2019–2023. https://doi.org/10.48420/21687809; Curious Travellers Editions: edited by Alex Deans and Ffion Mair Jones, University of Wales, project funded by the AHRC, https://curioustravellers.ac.uk/en/; Electronic Enlightenment, https://www.e-enlightenment.com/

13 Sarah Goldsmith, *Masculinity and Danger on the Eighteenth-Century Grand Tour* (London: Royal Historical Society, 2020), 129–30.

14 J. A. Chartres, ‘Road Carrying in England in the Seventeenth Century: Myth and Reality’, *Economic History Review*, 30 (1977), 78.

15 Brian Austen, ‘The Impact of the Mail Coach on Public Services in England and Wales, 1784–1840’, *Journal of Transport History*, 2 (1981), 26–28.

16 Dorian Gerhold, ‘The Development of Stage Coaching and the Impact of Turnpike Roads, 1653–1840’, *The Economic History Review*, 67 (2014), 821.

17 Charles Knight, *Once Upon a Time* (London: John Murray, 1859), 122–23.

18 Jackson, *Material Masculinities*, 136.

19 Jackson, *Material Masculinities*, 136. See also Ben Jackson, ‘The Thrill of the Chaise: Gendering the Phaeton in Eighteenth-Century Literary and Satirical Culture, c. 1760–1820’, in Jennifer Buckley, and Montana Davies-Shuck, eds, *Character and Caricature, 1660–1820* (London: Palgrave Macmillan, 2024), 142.

20 Castor Durante Da Gualdo, *A Treasure of Health By Castor Durante Da Gualdo, Physician and Citizen of Rome. Wherein is Shewn How to Preserve Health, and Prolong Life* (London: printed for William Crook, 1686), 11.

21 Gideon Harvey, *Morbus Anglicus: or, The Anatomy of Consumptions Containing the Nature, Causes, Subject, Progress, Change, Signes, Prognosticks, Preservatives* (London: printed for Nathaniel Brook, 1666), 222.

22 Letter, Reverend John Beale to Henry Oldenburg, 14 September 1665, available at https://doi-org.uoelibrary.idm.oclc.org/10.13051/ee:doc/boylroPC0020504a2c, accessed on 6 August 2025.

23 John Pechey, *A Collection of Chronical Diseases* viz*. The Colick, the Bilious Colick, Hysterick Diseases, The Gout, and The Bloody Urine from the Stone in the Kidnies[…]* (London: Printed by J.R., 1695), 69, 132.

24 For example, John Hill, *A Method of Curing the Jaundice and Other Disorders of the Liver* (London: printed by R. Baldwin, 1768), 25.

25 Thomas Coe, *A Treatise on Biliary Concretions: Or, Stones in the Gall-bladder and Ducts* (London: D. Wilson and T. Durham, 1757), 323.

26 Francis Fuller, *Medicina Gymnastica: or A Treatise Concerning the Power of Exercise […]* (London: Printed by John Matthews, 1705), 171.

27 Anon, ‘On the Curative Effects of Carriage and Travelling Exercise. By M. Coriolis; With Observations by Dr Johnson’, *The Medico-Chirurgical Review No. XIX, October 1 1828 to January 1 1829* (1829), 519.

28 Anon, ‘On the Curative Effects’, 520.

29 Edward Ward, *The London Spy, for the Month of May 1699, Part VII* (London: printed by J. Howe, 1702).

30 See, for example, Peter Templeman, *Select Cases, and Consultations, in Physick.* (London: L. Davis, 1757), 136. William Hay, *Deformity: An Essay* (Dublin: Printed by George Faulkner, 1754), 40.

31 Thomas Dyche and William Pardon, *A New General English Dictionary, Peculiarly Calculated for the Use and Improvement of such as are Unacquainted with the Learned Languages* (London, printed for Richard Ware, 1737), 424. See also Anon, *The Best and Easiest Method of Preserving Uninterrupted Health to Extreme Old Age* (London: Printed and Sold by R. Baldwin, 1748), 108, noting ‘the jolting of a coach’ as a form of ‘violent motion’.

32 Anon, *The British Apollo: Containing Two Thousand Answers to Curious Questions in Most Arts and Sciences…in Three Volumes, Volume Three* (London: Printed for Theodore Sanders, 1726), 715.

33 John E. Crowley, ‘The Sensibility of Comfort’, *The American Historical Review* (June 1999), 750–51.

34 Letter, Thomas Gray to Dr Thomas Wharton, 18 October 1753, available at https://doi-org.uoelibrary.idm.oclc.org/10.13051/ee:doc/graythOU0010385a1c, accessed on 18 August 2025.

35 Daniel Jütte, ‘The Stagecoach as Social Experiment: Gender, Class and the History of Travel’, *Journal of Tourism History*, 17 (2025), 351.

36 Jütte, ‘The Stagecoach as Social Experiment’, 352.

37 Letter, Arthur Murphy to James Murphy, 2 September 1747, available at https://doi-org.uoelibrary.idm.oclc.org/10.13051/ee:doc/murparEE0010002a1c, accessed on 18 August 2025. See Donald F. Bond, ed, *The Spectator, Volume 2* (Oxford: Oxford University Press, 1987), 439–40.

38 Gregory Smith, ed, *The Spectator in Four Volumes, Volume 2* (London: J.M. Dent, 1904), 273.3.

39 Daniel Jütte, ‘The Stagecoach as Social Experiment’, 351.

40 Letter from Jeremy Bentham to Sarah Wise, 13 December 1777, available at https://doi-org.uoelibrary.idm.oclc.org/10.13051/ee:doc/bentjeOU0020075a1c, accessed on 21 July 2023.

41 Letter, John Locke to John Locke Senior, 4 November 1656, available at https://doi-org.uoelibrary.idm.oclc.org/10.13051/ee:doc/lockjoOU0010041a1c, accessed on 6 August 2025.

42 Letter, David Hume to Charles Erskine, 24 February 1748, available at https://www-e-enlightenment-com.uoelibrary.idm.oclc.org/item/humedaOU0010111b1c/?srch_type=letters&all=coach&lang_main=all&r=425, accessed on 18 August 2025.

43 Robert Latham and William Matthews, eds, *The Diary of Samuel Pepys, Volume 7, 1666* (London: Harper Collins, 1971), 348.

44 Letter William Wycherley to Alexander Pope, 2 April 1706, available at https://doi-org.uoelibrary.idm.oclc.org/10.13051/ee:doc/popealOU0010014a1c, accessed on 7 August 2025.

45 Charlotte Campton, *The Economy of the Drinking House: Notions of Credit and Exchange in the Tavern in Early Modern English Drama* (University of Exeter: Unpublished PhD Thesis, 2014), 124.

46 Jütte, ‘The Stagecoach as Social Experiment’, 347.

47 Choi, ‘Every Jolt’, 143–44.

48 Choi, ‘Every Jolt’, 146, 148.

49 Andrew Wear, *Knowledge and Practice in English Medicine, 1550–1680* (Cambridge: Cambridge University Press, 2000), 190–91.

50 Boyle, *A Free Enquiry*, 320.

51 William Tullett, *Smell in Eighteenth-Century England: A Social Sense* (Oxford: Oxford University Press, 2019), 185–86.

52 See examples in Jütte, ‘Stagecoach as Social Experiment’, 351.

53 Samuel Smith, *The Bengal Almanac for the Year 1829* (Bengal Hurkaru and Chronicle Press, 1829), 32.

54 Letter from Jeremy Bentham to Samuel Parr, 16 September 1803, available at https://doi-org.uoelibrary.idm.oclc.org/10.13051/ee:doc/bentjeOU0070242a1c, accessed on 21 July 2023.

55 Letter Alexander Pope to Jonathan Swift, 2 September 1726, available at https://doi-org.uoelibrary.idm.oclc.org/10.13051/ee:doc/popealOU0020387b1c, accessed on 11 August 2025.

56 Letter, John Dickenson to Mary Hamilton, 19 June 1789, available at https://www.digitalcollections.manchester.ac.uk/view/AR-HAM-00001-00002-00015/1, accessed on 20 February 2026.

57 An Experienced Traveller, *Useful Instructions for Travellers,* (London: Printed for the author and sold by J. Owen, 1793), 11.

58
*Elizabeth Montagu, The Queen of the Bluestockings: Her Correspondence from 1762 to 1800*, ed. by Reginald Blunt, 2 vols, vol. 1 (London: John Murray, 1923), 24–25.

59 Letter Elizabeth Montague to Hester Thrale Piozzi, 13 October 1781, available at https://emco.swansea.ac.uk/emco/letter-view/1828/?q=passengers, accessed on 20 February 2026.

60 Sheffield Archives, Hare and Elliott Families of Sheffield, LD1576/7/14, letter J[enny] Brownsword to Ann Hare 17 August [c1785], 1781785 available at https://socialbodies.bham.ac.uk/database/letters/810/?tab=cite, accessed on 20 February 2026.

61 Letter, Alexander Mackenzie to Roderick Mackenzie, 14 January 1819, available at https://doi-org.uoelibrary.idm.oclc.org/10.13051/ee:doc/mackalCU0010522b1c, accessed on 14 August 2025.

62 Benjamin Motte, *The Philosophical Transactions from the Year M DCC (where Mr. Lowthorp ends) to the year M DCC XX. abridg'd, and dispos’d under general heads. In two volumes, Volume 1* (London: printed for R. Wilkin, R. Robinson, S. Ballard, W. and J. Innys, and J. Osborn, 1721), 105–106.

63 Letter Sir Samuel Bentham to Jeremy Bentham, 31 January 1784, available at https://doi-org.uoelibrary.idm.oclc.org/10.13051/ee:doc/bentjeOU0030239b1c, accessed on 14 August 2025.

64 Letter, Hester Lynch Piozzi to John Williams, 14 July 1819, available at https://doi-org.uoelibrary.idm.oclc.org/10.13051/ee:doc/piozheUD0060299a1c, accessed on 14 July 2025.

65 Letter Jeremy Bentham to Jeremiah Bentham, 23 September 1785, available at https://doi-org.uoelibrary.idm.oclc.org/10.13051/ee:doc/bentjeOU0030355a1c, accessed on 14 August 2025.

66 Isaac Cruikshank, ‘Symptoms of Jolting’ and ‘Jolting Preventatives’, in George Woodward, *Eccentric Excursions or Literary and Pictorial Sketches of Countenance, Character and Country in Different Parts of England and South Wales* (London: Allen and Co., 1796), 164–66. Images courtesy of The Lewis Walpole Library, Yale University.

67 Sophia Hoare, ‘Journal of a Tour into S. Wales in 1809’ available at editions.curioustravellers.ac.uk/doc/0028, accessed on 20 August 2025.

68 ‘Anne Lister's diary, tour of North Wales 11-27 July 1822’ transcribed with an Introduction by Kirsty McHugh and edited by Elizabeth Edwards available at editions.curioustravellers.ac.uk/doc/0023, accessed on 20 August 2025.

69 Letter, William Cowper to Samuel Rose, 7 August 1792, available at https://doi-org.uoelibrary.idm.oclc.org/10.13051/ee:doc/cowpwiOU0040166a1c, accessed on 24 August 2025.

70 Letter William Cowper to Catharine Courtenay-Throckmorton, 12 August 1792, available at https://doi-org.uoelibrary.idm.oclc.org/10.13051/ee:doc/cowpwiOU0040169a1c, accessed on 24 August 2025.

71 Letter, William Cowper to Reverend William Unwin, 3 July 1782, available at https://doi-org.uoelibrary.idm.oclc.org/10.13051/ee:doc/cowpwiOU0020061a1c, accessed on 20 August 2025.

72 Campton, *The Economy of the Drinking House*, 125.

73 Letter, Alexander Pope to Joseph Spence undated, 1733, available at https://doi-org.uoelibrary.idm.oclc.org/10.13051/ee:doc/popealOU0030380a1c, accessed on 14 August 2025.

74 Letter, Alexander Pope to Ralph Allen, 15 November 1740, available at https://doi-org.uoelibrary.idm.oclc.org/10.13051/ee:doc/popealOU0040290a1c, accessed on 14 August 2025.

75 Letter Alexander Pope to Ralph Allen, 28 November 1740, available at https://doi-org.uoelibrary.idm.oclc.org/10.13051/ee:doc/popealOU0040297a1c, accessed on 14 August, 2025.

76 Alexander Pope, *The Rape of the Lock*, Canto 1, line 96.

77 Letter, Benjamin Franklin to Dr Richard Price, 16 August 1784, available at https://doi-org.uoelibrary.idm.oclc.org/10.13051/ee:doc/pricriDU0020224a1c, accessed on 14 August 2025.

78 Jackson, *Material Masculinities*, 133.

79 Advertisement, ‘Edmond Hammond at the Boot Inn’, *Saunders’s News Letter*, 7 February 1783, 2.

80 Thomas Fuller, *An Essay on Wheel Carriages* (London: Longman, Rees, Orme, Brown and Green, 1828), 19, 21. See Also, William Felton, *A Treatise on Carriages* (London: J. Debrett, 1794), 234.

81 L. Herbert, ed, *The Register of Arts and Journal of Patent Inventions,* 4th edn (London: B. Steill, 1830), 227.

82 Anon, *The British Apollo: Containing About Two Thousand Answers to Curious Questions in Most Arts and Sciences, Serious, Comical, and Humorous, Approved of by Many of the Most Learned and Ingenious of Both Universities, and of the Royal Society* (London: printed for John Isted, 1718), 393.

83 Jones, *The mysteries of opium reveal'd,* 241.

84 See, for example, Henrietta Rouviere Moss, *Gratitude and Other Tales, In Three Volumes, Volume 1* (London: A.K. Newman and Co., 1826), 42.

85 Doreen Huppert et al., ‘A Historical View of Motion Sickness—A Plague at Sea and on Land, Also with Military Impact’, *Frontiers in Neurology*, 8 (2017), 1.

86 Huppert et al, ‘A Historical View’, 1.

87 Hugh Chamberlain, *Manuale Medicum: or a Small Treatise of the Art of Physick in General, and of Vomits and the Jesuits Powders in Particular* (London: Printed by J. Gain, 1685), 28.

88 Chamberlain, *Manuale Medicum,* 27.

89 Nicholas Culpeper, Abdiah Cole and William Rowland, *The Practice of Physick in Seventeen Several Books…Being Chiefly a Translation of the Works of That Learned and Renowned Doctor Lazarus Rivierus* (London: Printed by Peter Cole, 1695), 295.

90 Ulinka Rublick, trans. Pamela Selwyn, ‘Fluxes: The Early Modern Body and the Emotions’, *History Workshop Journal*, 53 (2002), 1.

91 Strother, *Practical Physician for Travellers,* 182.

92 Strother, *Practical Physicial for Travellers*, 182.

93 Strother, *Practical Physician for Travellers*, 182.

94 G. Havers and J. Davies (trans), *Another Collection of Philosophical Conferences of the French Virtuosi, Upon Questions of All Sorts; For the Improving of Natural Knowledge* (London: Printed for Thomas Dring and J. Starkey, 1665), 97.

95 Thomas Willis, *Pharmaceutice rationalis: or, An exercitation of the operations of medicines in humane bodies* (London: printed for Thomas Dring et al., 1678), 24.

96 Michael Etmuller, *Etmullerus Abridg'd: or, A Compleat System of the Theory and Practice of Physic. Being a Description of All Diseases Incident to Men, Women and Children* (London: Printed for E. Harris, 1699), 84–85.

97 John Jones, *The Mysteries of Opium Reveal’d, by Dr John Jones…*(London: Printed for Richard Smith, 1700), 241.

98 Havers and Davies, *Another Collection,* 97.

99 Havers and Davies, *Another Collection*, 97.

100 Jackson, ‘Thrill of the Chaise’, 144–45.

101 William Okely, *Pyrology; or, the connexion between natural and moral philosophy: with a short disquisition on the origin of Christianity* (London: printed for J. Johnson, 1797), 99.

102 Okely, *Pyrology*, 101.

103 Letter Jeremy Bentham to Jeremiah Bentham, 26 September 1767, available at https://doi-org.uoelibrary.idm.oclc.org/10.13051/ee:doc/bentjeOU0010120a1c, accessed on 18 August 2025.

104 British Library MS ADD 35183 p. 25 (document 13). Letter from Gilbert White (at Selborne) to Pennant (no address given) 28 February 1769.

105 Robert Boyle, *A Free Enquiry into the Vulgarly Receiv'd Notion of Nature Made in an Essay Address’d to a Friend* (London: Printed by H. Clark, 1686), 319–20.

106 Okely, *Pyrology*, 101.

107 Adam Fitzadam, *The World*, XXXVII, 13 September 1753, 223.

108 Sir John Narborough, *An Account of Several Late Voyages & Discoveries to the South and North Towards the Streights of Magellan, the South Seas, the Vast Tracts of Land Beyond Hollandia Nova* (London: Printed for Sam. Smith, 1694), 33.

109 Samuel Johnson, *A Dictionary of the English Language…In Two Volumes, Volume 2, second edition* (London: printed for W. Strahan, 1755).

110 John Griffith, *A Journal of the Life, Travels, and Labours in the Work of the Ministry, of John Griffith* (London: printed by James Phillips, 1779), 271.

111 Jonas Hanway, *A Journal of Eight Days Journey from Portsmouth to Kingston Upon Thames…* (London: printed by H. Woodfall, 1756), 86.

